# Supplementary material for: Current-induced domain wall motion in a van der Waals ferromagnet Fe3GeTe2
Source: Nat Commun. 2024 Jun 6;15:4851. doi: 10.1038/s41467-024-48893-y (PMC11156869; doi:10.1038/s41467-024-48893-y)
Supplement: Supplementary file 1 — Supplementary Information [file 41467_2024_48893_MOESM1_ESM.pdf]

# Supporting Information

# Current-induced domain wall motion in a van der Waals ferromagnet $Fe_3GeTe_2$

## Table of Contents

Characterization of $Fe_3GeTe_2$ devices ........ 2
Electrical resistance of a $Fe_3GeTe_2$ device........ 3
Dependence of current-induced domain wall motion........ 4
Temperature dependence of the MOKE contrast........ 5
MOKE images of the CIDWM ........ 6
Ultra high vacuum transfer system........ 7
Fitting parameters for 1D-model of current induced domain wall motion ........ 8
Reference ........ 9

# Characterization of $Fe_3GeTe_2$ devices

Figure S1 shows the optical image of the measured device in Figure 1 of the main text.

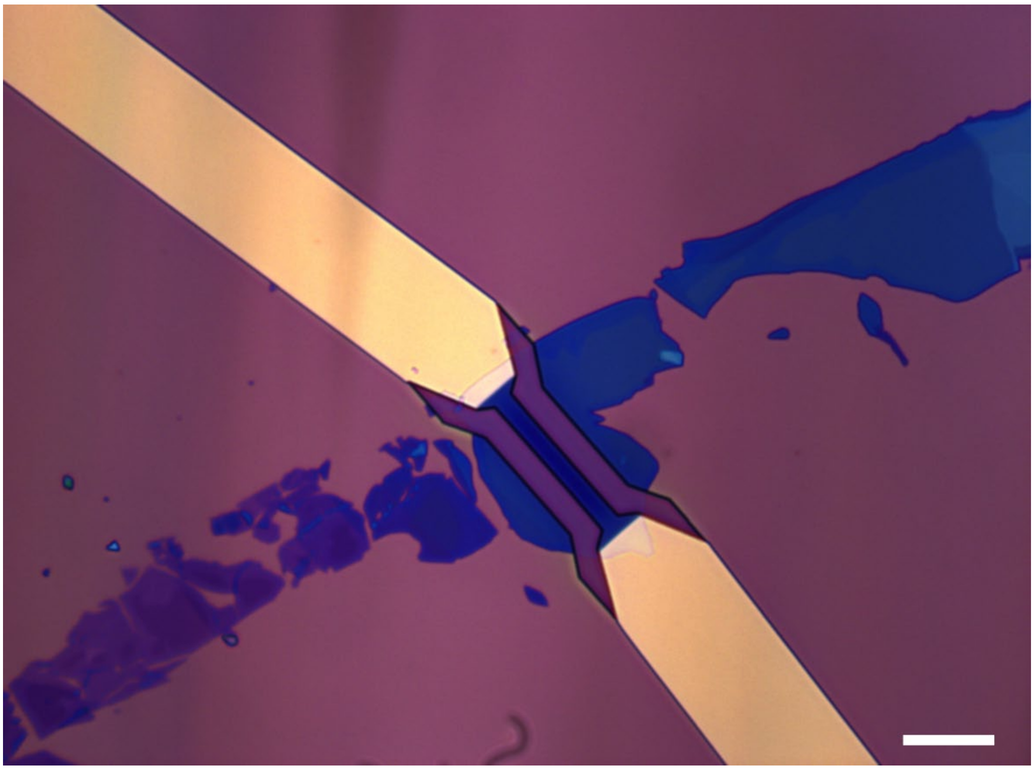

**Figure S1. Optical image of the device measured in Figure 1.** The scale bar is 10 μm. The gold colored regions correspond to the electrical contacts. The flake is shown in blue. A rectangular region was defined in the flake by Ar ion milling. The purple regions correspond to the $SiO_2$.

# Electrical resistance of a $Fe_3GeTe_2$ device

The electrical resistance of the $Fe_3GeTe_2$ device in Figure 1 was measured by a two-probe method from 20 K to 160 K. The resistivity was calculated from measurements of the device dimensions. The resistivity varied by less than ~7% over the measured temperature range from 20 K to the Curie temperature (~160 K). Therefore, the resistance is treated as a constant unchanged in the fit shown in the inset of Figure 1e. The resistivity is similar to values reported in the literature. [1]

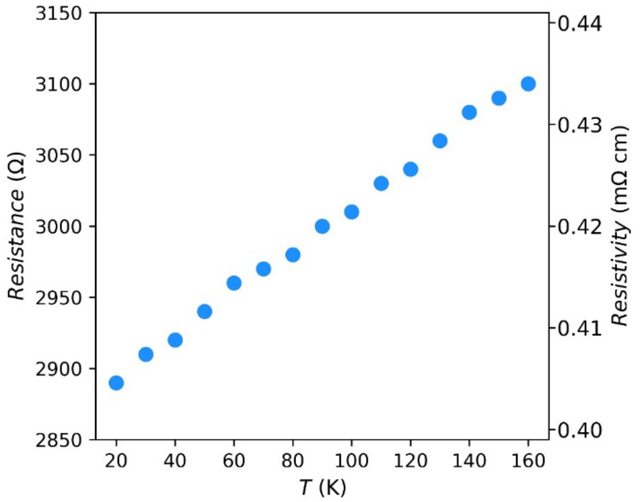


**Figure S2. Temperature dependence of the resistance and resistivity of the $Fe_3GeTe_2$ device in Figure 1.**

## Dependence of current-induced domain wall motion

Figure S3 shows the longitudinal field dependence of domain wall motion with and without DMI fitted by 1D analytical model [2]. In the presence of DMI, in-plane field dependence of up/down and down/up domain wall processes none-zero peaks, which is related to the effective DMI field. On the contrary, in the absence of DMI, two types of domain wall show the same dome-like dependence. In figure S4, velocities in FGT with different thicknesses, present similar temperature dependence.

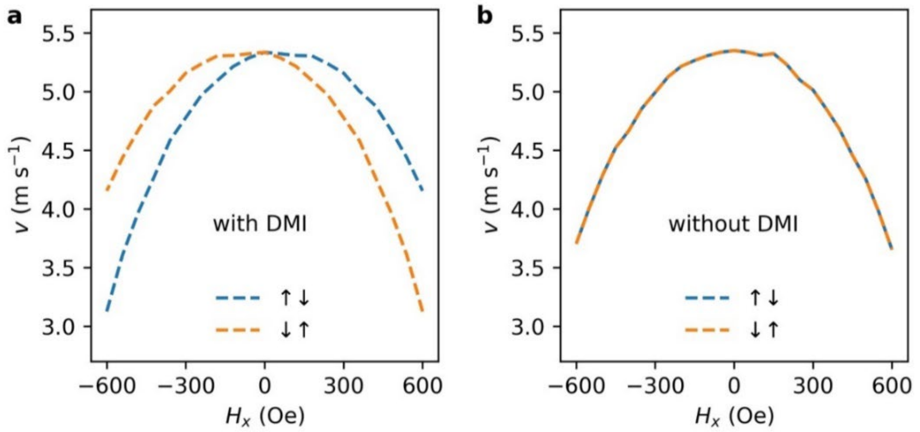


**Figure S3. Longitudinal field dependence of DW velocities from the simulation based on 1D analytical model.** a, with DMI and b, without DMI.

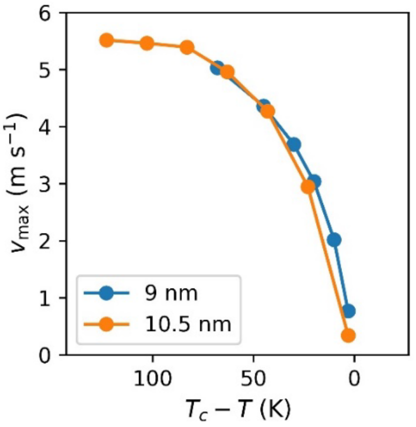


**Figure S4. Thickness dependence of velocity of CIDWM in pristine FGT**. Orange curve shows the velocity in a sample with thickness of 10.5 nm. Blue curve shows the velocity in another sample with thickness of 9 nm. Due to the different $T_c$ of two samples, curves are normalized as $T_c$-$T$ versus $v_{max}$.

## Temperature dependence of the MOKE contrast

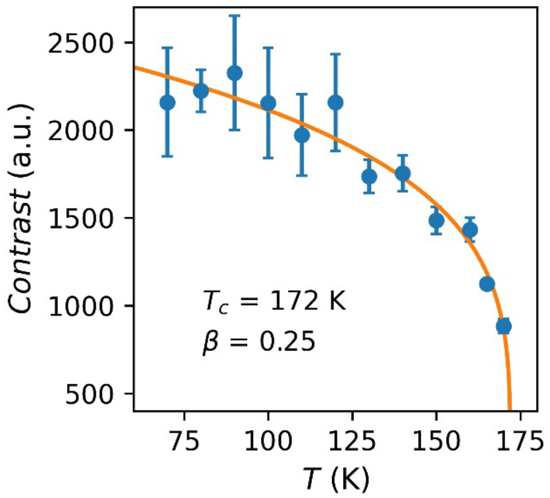


**Figure S5. Temperature dependence of the MOKE contrast of a 9 nm FGT flake**. Data are fitted by the critical power-law form $(1\text{-}T/T_c)^{\beta}$, where $T_c$ is 172 K and $\beta$ is 0.25[3]. Error bars represent the difference between the maximal value and the minimum value.

# MOKE images of the CIDWM

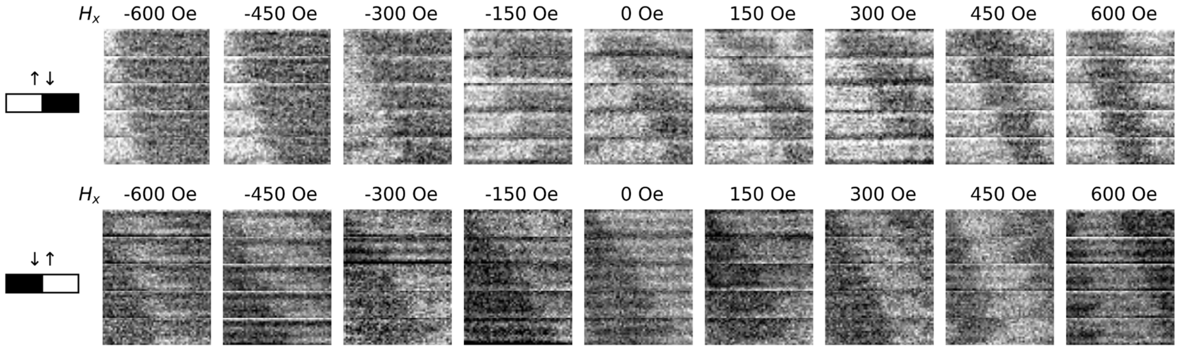


**Figure S6. MOKE images of the CIDWM under various longitudinal fields of a 9 nm FGT flake at 80 K.**

# Ultra high vacuum transfer system

Figure S7 shows the picture of our ultra-high vacuum transfer system. $Fe_3GeTe_2$ flakes are exfoliated first in the glovebox (highlighted with the blue box) and then are transferred through an ultra-high vacuum tube (highlighted with the green box) to the magnetron sputtering system (highlighted with the yellow box) for deposition of platinum or tungsten.

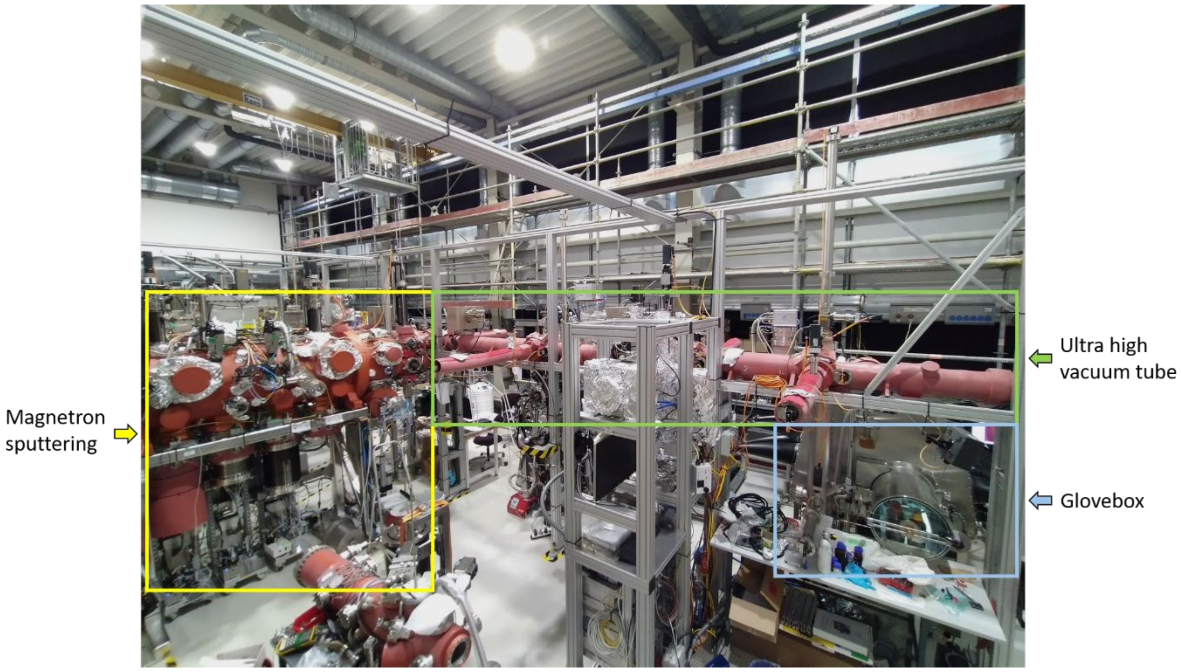


**Figure S7. Ultra high vacuum tube system that connects the ultra-high vacuum magnetron sputtering system and the glovebox.**

## Fitting parameters for 1D-model of current induced domain wall motion

| | Pristine FGT | FGT/W |
|---|---|---|
| $\alpha$ | 0.62 | 0.8 |
| $\beta$ | 0 | 0 |
| $\Delta$ (Å) | 3.2 | 3 |
| $H_{\mathrm{k}}$ (Oe) | 300 | 450 |
| $H_{\mathrm{SHE}}$ (Oe) | 0 | 25 |
| $u$ (m $s^{-1}$) | 7.5 | 6.7 |
| $H_{\mathrm{DMI}}$ (Oe) | 70 | 140 |
| $M_{\mathrm{S}}$ (emu $cm^{-3}$) | 350 | 350 |

**Table S1. List of fitting parameters for the fits in Fig. 4 of the main text.** A 1D DW model for describing the current induced domain wall motion with the presence of both STT and SOT is used as described in *Filippou et al. 2018* is used[4].

# Reference


1. Chen, B., et al., *Magnetic Properties of Layered Itinerant Electron Ferromagnet Fe3GeTe2.* Journal of the Physical Society of Japan, 2013. **82**(12): p. 124711.
2. Yang, S.-H., K.-S. Ryu, and S. Parkin, *Domain-wall velocities of up to 750 m s−1 driven by exchange-coupling torque in synthetic antiferromagnets.* Nature Nanotechnology, 2015. **10**(3): p. 221-226.
3. Fei, Z., et al., *Two-dimensional itinerant ferromagnetism in atomically thin Fe3GeTe2.* Nature Materials, 2018. **17**(9): p. 778-782.
4. Filippou, P.C., et al., *Chiral domain wall motion in unit-cell thick perpendicularly magnetized Heusler films prepared by chemical templating.* Nature Communications, 2018. **9**(1): p. 4653.
